# Short- and long-term outcomes of pulmonary exposure to a sublethal dose of ricin in mice

**DOI:** 10.1038/s41598-024-62222-9

**Published:** 2024-05-21

**Authors:** Anita Sapoznikov, Yentl Evgy, Amir Ben-Shmuel, Arieh Schwartz, Ron Alcalay, Moshe Aftalion, Alon Ben David, Noam Erez, Reut Falach

**Affiliations:** 1 Department of Biochemistry and Molecular Genetics, Israel Institute for Biological Research, 74100 Ness-Ziona, Israel; 2 Department of Infectious Diseases, Israel Institute for Biological Research, 74100 Ness-Ziona, Israel; 3 Department of Biotechnology, Israel Institute for Biological Research, 74100 Ness-Ziona, Israel

**Keywords:** Animal disease models, Cytokines, Immunochemistry, Respiratory distress syndrome, Inflammation

## Abstract

Ricin, an extremely potent toxin produced from the seeds of castor plant, *Ricinus communis*, is ribosome-inactivating protein that blocks cell-protein synthesis. It is considered a biological threat due to worldwide availability of castor beans, massive quantities as a by-product of castor oil production, high stability and ease of production. The consequence of exposure to lethal dose of ricin was extensively described in various animal models. However, it is assumed that in case of aerosolized ricin bioterror attack, the majority of individuals would be exposed to sublethal doses rather than to lethal ones. Therefore, the purpose of current study was to assess short- and long-term effects on physiological parameters and function following sublethal pulmonary exposure. We show that in the short-term, sublethal exposure of mice to ricin resulted in acute lung injury, including interstitial pneumonia, cytokine storm, neutrophil influx, edema and cellular death. This damage was manifested in reduced lung performance and physiological function. Interestingly, although in the long-term, mice recovered from acute lung damage and restored pulmonary and physiological functionality, the reparative process was associated with lasting fibrotic lesions. Therefore, restriction of short-term acute phase of the disease and management of long-term pulmonary fibrosis by medical countermeasures is expected to facilitate the quality of life of exposed survivors.

## Introduction

Ricin toxin is a type II ribosome-inactivating protein easily produced from the castor bean plant *Ricinus communis*. The high potency, extremely small lethal dose, ease of preparation, stability and global availability of the castor bean plant make ricin a potential bioterror agent. Accordingly, it is considered to be a high-risk chemical for living beings under the Organization for the Prohibition of Chemical Weapons^1^ and is also classified as a Category B biological agent by the Centers for Disease Control and Prevention^2^. The catalytic activity of ricin is characterized by the cleavage of the 28S rRNA subunit, resulting in termination of protein synthesis and the consequent cell death^3^. Previous studies in rodents and non-human primates demonstrated that following pulmonary exposure to lethal dose of ricin, the injury is mostly confined to the lungs, including marked interstitial pneumonia associated with pro-inflammatory cytokine release, massive neutrophil infiltration, vascular hyperpermeability, perivascular and alveolar edema, hemorrhages, diffuse airway epithelial cell and alveolar macrophage death. Eventually, the extensive pneumonia, which involves massive cell infiltration and excessive accumulation of pleural fluids leads to respiratory insufficiency and death^4–20^. This damage to the lungs is classified as acute lung injury, which can develop to acute respiratory distress syndrome (ARDS)^12^. Although the consequences of pulmonary ricinosis have been extensively investigated in the past, the data on the effect of sublethal ricin intoxication beyond the acute stage is limited. Since it is assumed that in an event of intentional ricin spread, majority of individuals will be exposed to sublethal rather than to lethal doses of the toxin^6^, there is a need to evaluate the short- and long-term effects, in order to understand the consequences of such exposure. Past studies have shown that in a murine model of sublethal challenge with ricin, mice were analyzed only during the first week following ricin exposure. They lost weight and histopathological analysis of their lungs showed alveolar edema, accompanied by the infiltration of inflammatory cells, hemorrhages in the first few days, and later, hyperplasia of bronchiolar and alveolar epithelial cells and collagen deposition^15,21,22^. In different rat models, inhalational sublethal exposure led to development of diffuse necrotizing pneumonia of the airways 2 days post exposure, alveolar macrophage and type II pneumocyte hyperplasia and interstitial fibrosis 7 days post ricin exposure^20,23^. Evaluation of ricin toxicity following sublethal aerosolized exposure in macaques was performed at 11- and 20-days post exposure. In these models, late pathological consequences included extensive fibrosis, pneumocyte hyperplasia and infiltrates of foamy macrophages within alveolar spaces were detected^6,10^. However, all the above-mentioned studies have not evaluated both the short-term damage and the long-term sequelae following sublethal ricin intoxication in a single study using the same animal model, the same ricin dose and the same route of exposure. Thus it is difficult to evaluate the effect of sublethal ricin exposure and to conclude whether this sublethal exposure causes impairment in daily function and long-term quality of life of surviving animals.

In the current study we established a mouse model for sublethal ricin intoxication. Using this model, we evaluated the short- and long-term effects of the toxin on various physiological disease parameters, such as voluntary activity and respiratory functions. We also characterized the tissue damage in the lung and the immune response changes that occurred following exposure. Our findings may assist in formulation of effective therapeutic strategies for alleviating the acute phase pathologies and long-term persistent pulmonary fibrosis.

## Results

### Physiological alterations following sublethal ricin exposure

**Figure 1:**
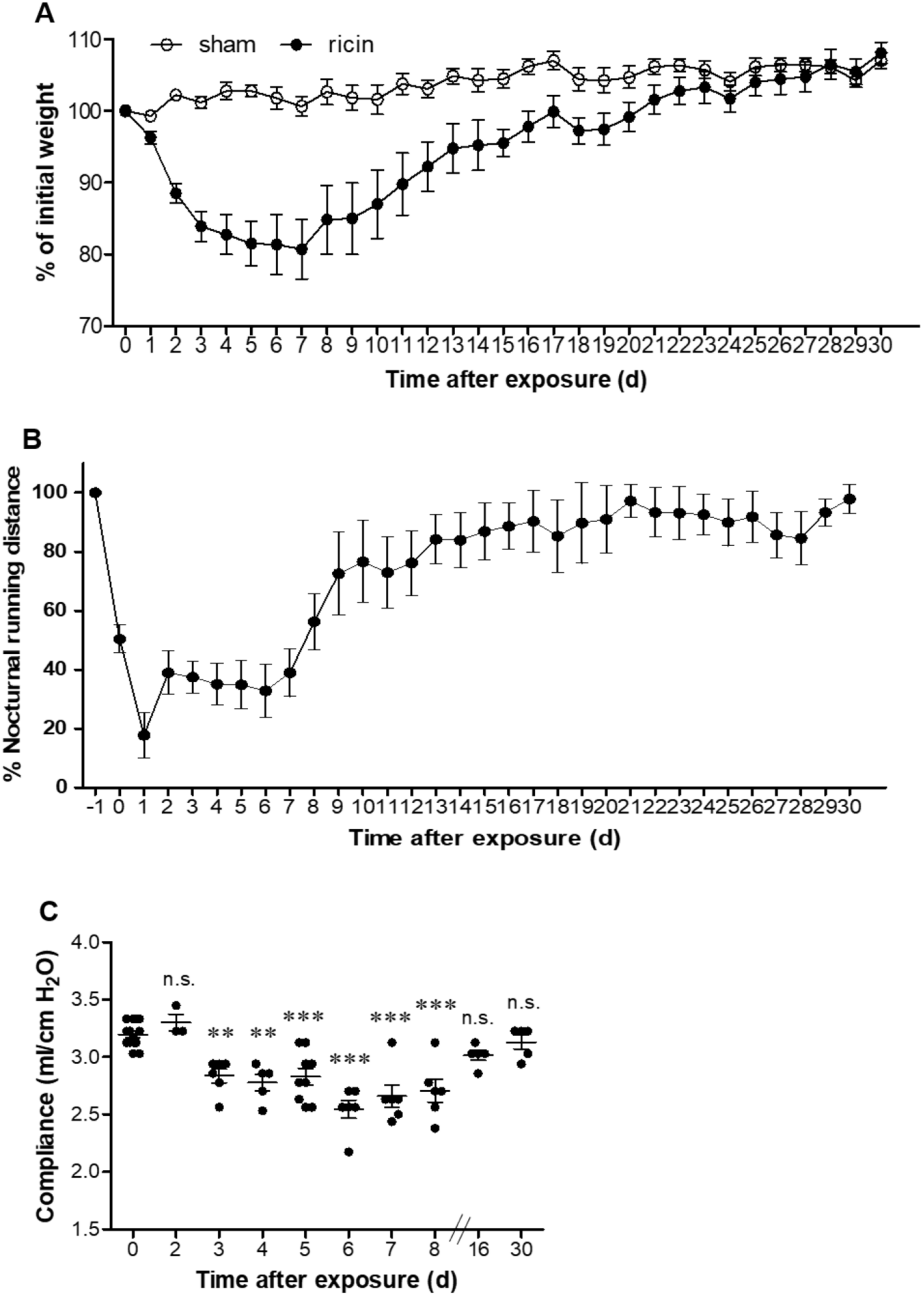
Physiological and functional changes in mice following sublethal exposure to ricin toxin. Mice were intranasally intoxicated with ricin (1.7 µg/kg body weight) and monitored for 30 days. (**A**) Morbidity as depicted by body weight loss following intoxication (*n* = *10*). (**B**) Activity profile of mice according to voluntary wheel running. Base line was determined as the average running distance of each animal from 5 days prior to intoxication (represented as 100% on day −1). Then, the mice were exposed to ricin (day 0) and their activity was further recorded for 30 days (*n* = *11*). (**C**) Lung compliance of ricin intoxicated mice over 30 days. Each dot represents one animal. Data are represented as means ± SEM. **P < 0.001, ***P < 0.0001, n.s., not significant, in comparison to non-intoxicated mice.

To characterize the physiological and pathological outcome of sublethal exposure of mice to ricin, we intoxicated mice with the maximal dose of ricin that did not result in mortality of mice (more than *n* = 50 mice^24^). To this end, mice were intranasally exposed to sublethal dose of ricin (1.7 µg/kg) equivalent to 0.35LD_50_. Using this model, we were able to examine the short- and long-term effects of pulmonary exposure to a sublethal dose of ricin, in which all animals survived the intoxication. Morbidity caused by the toxin was first characterized by weight loss. Following sublethal exposure to ricin, mice lost weight gradually and continuously, reaching maximal weight loss of ~ 20% at day 5–7 post exposure. According to this finding, day 6 post exposure was selected as the time-point of maximal morbidity. At later time-points, there was gradual increase in body weight and at day 30 mice reached their initial body weight, prior to ricin intoxication (Fig. 1A). According to this weight follow up, we could segregate the evaluation of the effects of sublethal intoxication into two different stages. The short-term damage at the peak of the disease (~ day 6) and the long-term damage (~ day 16 and 30). Next, we examined the impact of sublethal ricin intoxication on the functional capacity of intoxicated mice by monitoring their nocturnal activity, which was measured by running distance on a wheel. The use of high-resolution monitoring of voluntary running wheel^25^ to access behavior and physical changes in rodents is common and can yield important information regarding disease state^26^. The monitoring of mouse voluntary running began 5 days prior to ricin intoxication to determine individual, characteristic and consistent running profile of each naïve mouse. During this time period, prior to intoxication, naïve mice ran consistently (SD ~ 7–10 km/night). Immediately after ricin intoxication, activity dramatically decelerated (Fig. 1B) and even completely halted for few days in some mice (Supplementary Fig. 1A–D). After a period of 6–7 days of minimal running activity, mice gradually returned to their normal pre-intoxication running pattern. Approximately 2 weeks after ricin intoxication, mice returned to their characteristic running pattern as was acquired prior to ricin intoxication (Fig. 1B). On average, animals returned to normal running activity, at day 11 ± 3 days post exposure to ricin.

In addition to measurements of mouse activity, we examined the compliance of the respiratory system. Lung compliance is frequently used to characterize respiratory diseases and provides information about the intrinsic elastic properties of the respiratory system^27^. As can be seen (Fig. 1C) lung compliance decreased significantly already 3 days following ricin intoxication and continued gradually to decline till day 6 post exposure with the maximal drop from 3.2 ± 0.1 ml/cm H_2_O (in healthy mice) to 2.5 ± 0.2 ml/cm H_2_O (in intoxicated mice at day 6). In a similar manner to the behavior pattern in running activity, lung compliance has recovered at later stages until complete restoration of airway functionality approximately 2 weeks after ricin intoxication.

Taken together, the measurements of body weight, running activity on the wheel and airway compliance were consistent and correlated with each other, providing us a precise understanding of the disease progression and segregation between the stages of the disease. Following sublethal ricin intoxication, there was escalation of disease severity during the first 6 days, which allowed us to determine the short-term pathology. This stage was followed by two weeks recovery phase, and later time-points (represented by day 16 and day 30) that allowed us to understand and examine the presence of long-term damage. Altogether, the measured physiological parameters showed that although mice experienced prominent injury, which was manifested by decreased movement, lung injury and diminished body weight, they gradually recovered and within a month following intoxication, the mice displayed normal functional capacity.

In order to determine whether exposure to a higher, yet still sublethal dose would result in disease with similar characteristics, we examined a slightly higher dose of ricin in order to understand whether there will be long-term effects among mice that survived ricin exposure. To this end, we intranasally exposed mice to a concentration of 2.4 µg/kg body weight, which represents 0.5LD_50_. We observed that with this partially lethal dose, 30% of the animals succumbed to the intoxication (Supplementary Fig. 2A) and surviving animals exhibited gradual weight loss reaching a minimum body weight (< 80% of initial body weight) on days 6–9 post intoxication (Supplementary Fig. 2B). In a similar manner to animals that were exposed to 0.35LD_50,_ also in this case lung compliance decreased on peak of the disease (day 7 post exposure). Importantly, during the recovery period, no significant changes were found in the compliance between surviving- and healthy animals (days 16 and 30, Supplementary Fig. 2C) even after exposure to this higher dose.

### Pathological examination of mice following sublethal ricin intoxication

**Figure 2:**
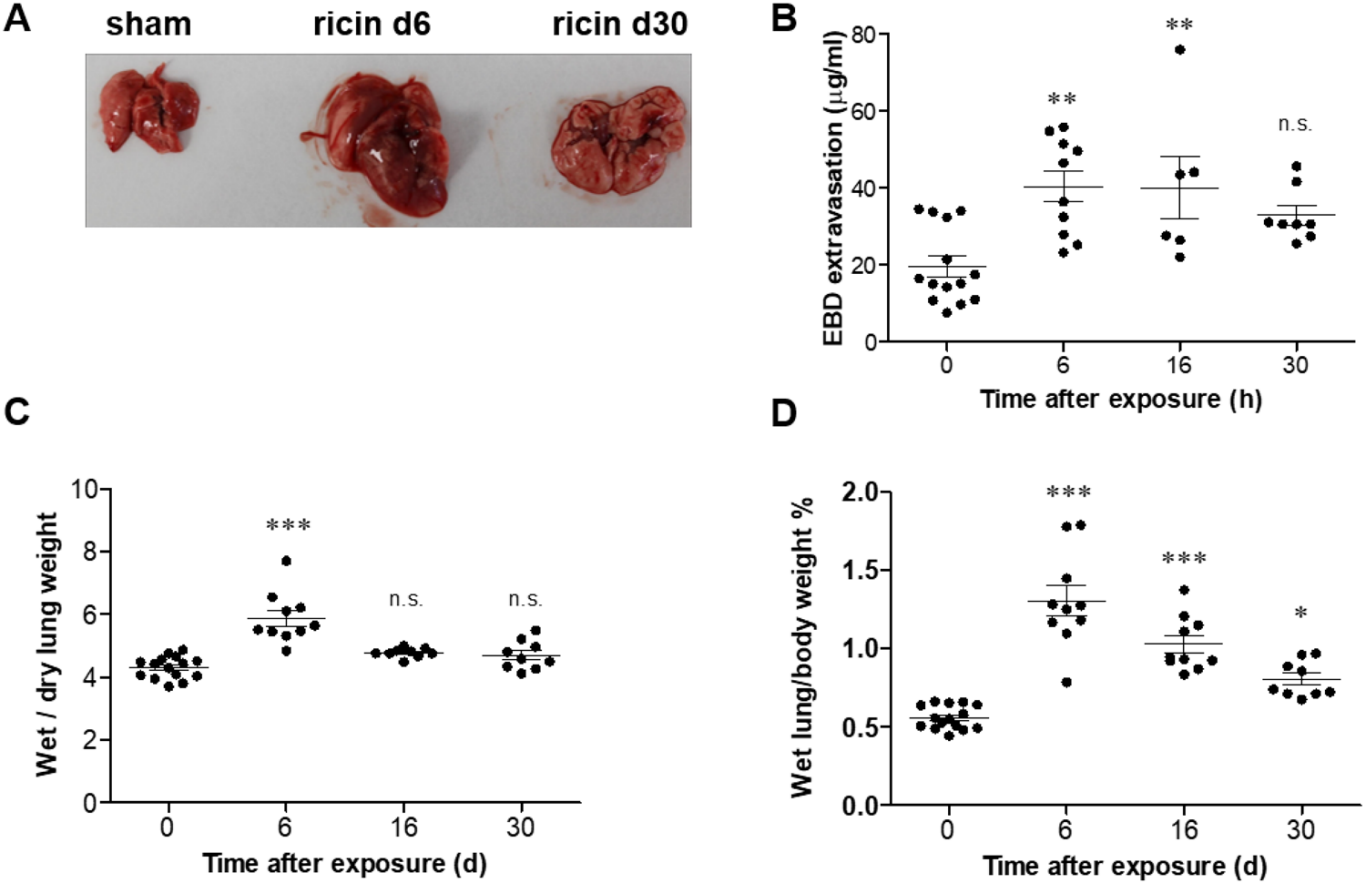
Lung permeability in mice following sublethal ricin intoxication. Mice were exposed to ricin intoxication (1.7 µg/kg body weight) by intranasal instillation. At different time points post exposure animals were sacrificed and their lungs were collected for analysis. (**A**) Lung morphology in control (sham) mice or intoxicated mice on day 6 or 30 post exposure. (**B**) Lung EBD analysis following ricin intoxication. Mice were intravenously injected with 50 mg/kg EBD at the indicated time points and lungs were harvested 1 h later for determination of EBD content. (**C**) Lung wet/dry weight ratio analysis following ricin intoxication. (**D**) Wet lung/body weight ratio analysis following ricin intoxication. In all figures each dot represents one animal and data are means ± SEM. *P < 0.05, **P < 0.01, ***P < 0.001 in comparison to non-intoxicated mice.

To evaluate the extent of lung injury in mice at different stages of the developed disease following sublethal ricin exposure, we examined the lungs of mice at different time-points post exposure. A morphological analysis of the whole lung showed that 6 days following exposure, all lobes of the lungs were grossly enlarged and hemorrhagic. At day 30, although lungs dimensions were reduced, they had not regained their normal morphology. At this late time point, lungs were still enlarged in comparison to healthy lungs, edematous and acquired a bright color, which is typical to fibrotic lungs (Fig. 2A). Since lethal pulmonary exposure to ricin results in the disruption of the epithelial and endothelial barriers, ensuing in increased permeability and decreased edema fluid clearance^7–9,11,12,28,29^, we tested the extent of edema and impairment of alveolar-capillary barrier integrity following sublethal toxin exposure. To assess the effect of sublethal dose of ricin on lung permeability, mice were intravenously injected with Evans Blue Dye (EBD) at different time points after intranasal exposure to ricin, lungs were harvested 1 h later and EBD was extracted and quantified. Pulmonary EBD levels were found to be elevated on day 6 and even on day 16 after ricin-intoxication (EBD values were approximately two-fold higher than measured in sham mice). Pulmonary EBD values determined at 30 days post-exposure, were reduced in comparison to the acute phase (day 6). However, they were still higher than the values of naïve mice, although not statistically different (Fig. 2B). In addition to EBD, the appearance of lung permeability and edema was confirmed by measurement of wet/dry lung weight and lung /body weight ratios. Both ratios were increased significantly at day 6 after ricin exposure (Fig. 2B,C), however, although wet/dry lung weight ratio returned to normal at later time-points (Fig. 2C), the lung/body weight ratio was still significantly high at days 16 and 30 after intoxication (Fig. 2D). This finding correlated with the abnormal lung morphology at this time-point (Fig. 2A). These results demonstrate that the lungs underwent pathological alveolar-capillary barrier impairment during the acute phase of the disease. This impairment did not completely alleviate and acquired long-term persistence.

**Figure 3:**
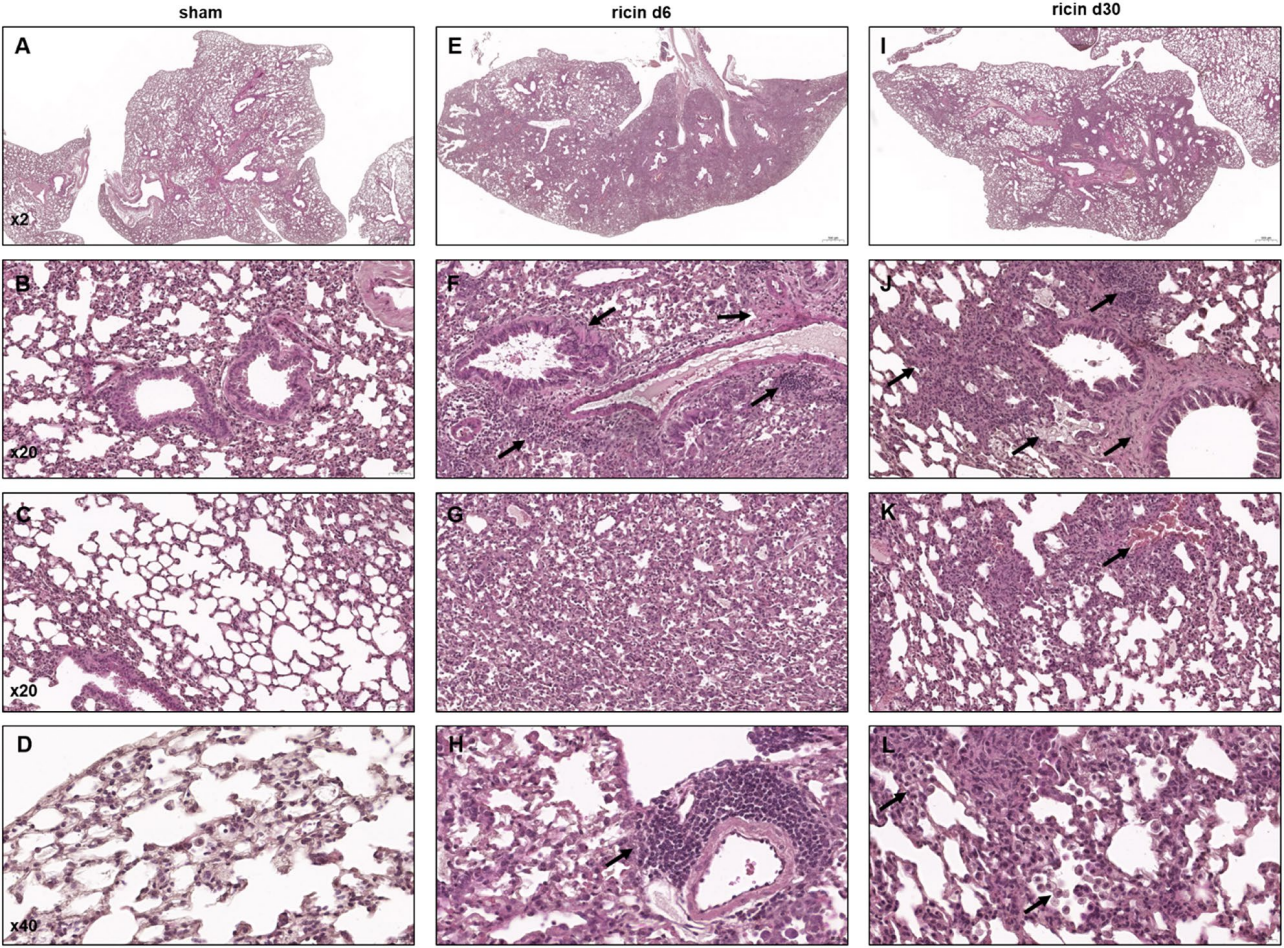
Histopathological lesions in mice following sublethal ricin intoxication. Paraffin-embedded lung tissues from mice 6 or 30 days following intranasal ricin intoxication (1.7 µg/kg body weight). Lung histopathology from control mice that were intranasally exposed to PBS exhibited normal lung architecture (**A–D**). Lung tissues from mice 6 days following ricin exposure (**E–H**). Lung tissues from mice 30 days following ricin exposure (**I–L**). Black arrows indicate the presented lesions. Panels are representatives of 5 mice in each group, scale bar: 500 µm (**A**,**E**,**I**); 50 µm (**B**,**C**,**F**,**G**,**J**,**K**); 20 µm (**D**,**H**,**L**).

To further characterize the ricin-inflicted injury of the lung tissue, H&E- stained sections prepared at different time-points following sublethal intoxication, were analyzed. In comparison to the intact healthy lungs (Fig. 3A–D), prominent and extensive pathological lesions were observed during the acute phase of the disease (day 6) (Fig. 3E). These lesions included widespread pulmonary edema, perivascular expansions with protein exudates and infiltrating cells, pronounced bronchiolitis and inflammation (Fig. 3F,G), fibrin accumulation in the alveolar airspaces, focal alveolar damage (Fig. 3G) and pulmonary neutrophil influx (Fig. 3H). Interestingly, during the recovery phase, at day 30 following ricin exposure, pulmonary lesions were still presented, albeit the damage was less widespread and focal (Fig. 3I). The damaged areas were characterized by focal perivascular and peribronchial edema, infiltration of inflammatory cells (Fig. 3J), hemorrhagic areas (Fig. 3K) and pronounced alveolar infiltration of giant macrophages with foamy cytoplasm (alveolar histiocytosis) (Fig. 3L).

**Figure 4:**
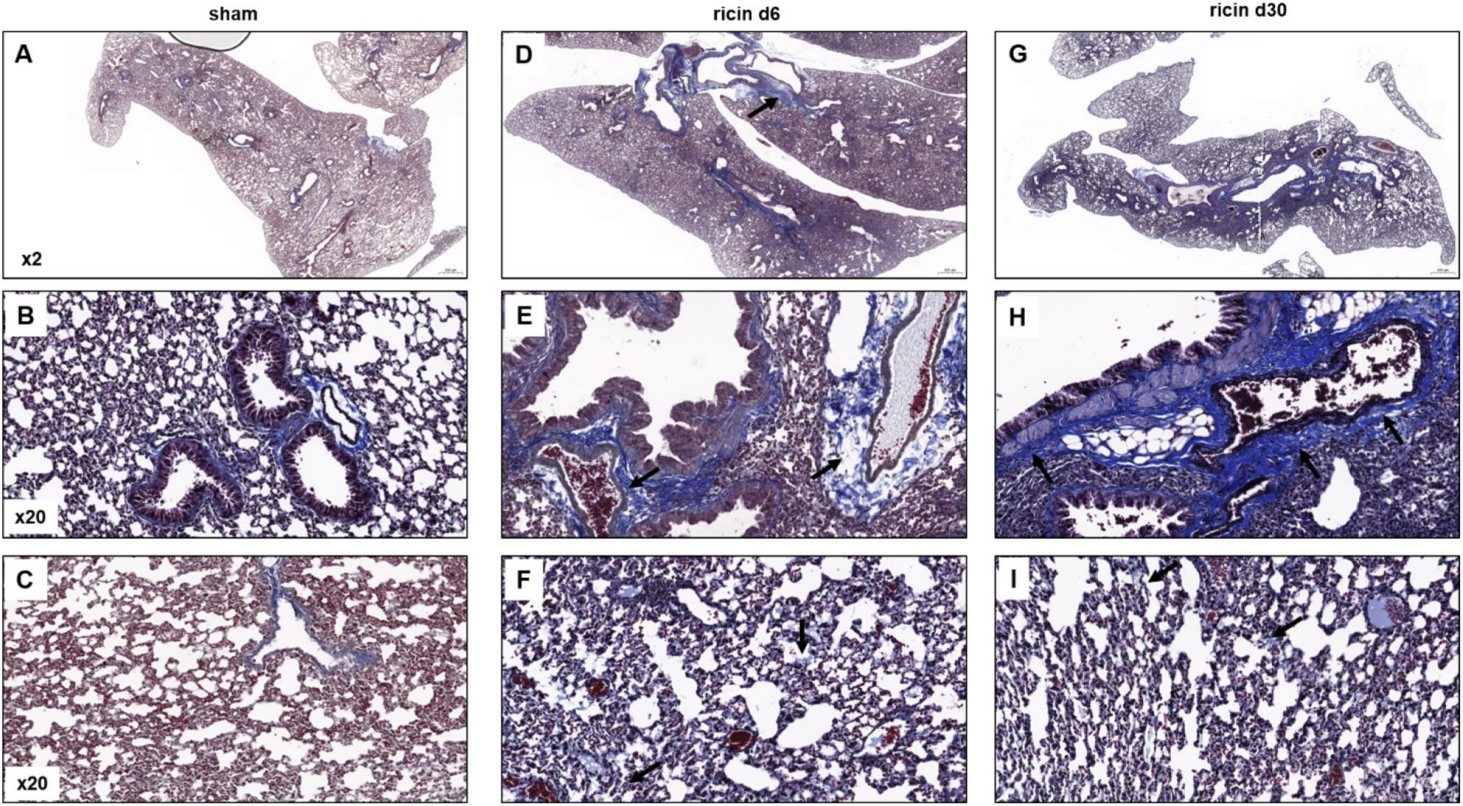
Collagen deposition in the lungs of mice following sublethal ricin intoxication. Mice were intranasally intoxicated with (1.7 µg/kg body weight) ricin, lungs were harvested at day 6 or 30 following exposure and Masson’s Trichrome staining was conducted on paraffin-embedded sections. Collagen deposition and fibrous connective tissue indicated in blue. (**A–C**) normal lung architecture in sham control lung intranasally exposed to PBS. (**D–F**) Lung sections from mice 6 days following ricin exposure. (**G–I**) Lung sections from mice 30 days following ricin exposure. (**A**,**D**,**G**) Low magnification (×2) of a lung lobes. (**B**,**E**,**H**) Bronchioles and blood vessel areas. (**C**,**F**,**I**) Alveoli. Black arrows indicate the presented lesions. Panels are representatives of 5 mice in each group, scale bar: 500 µm (**A**,**D**,**G**); 50 µm (**B**,**C**,**E**,**F**,**H**,**I**).

Collagen deposition analysis, by Masson’s trichrome staining, showed abundant collagen accumulation and fibrotic process (Fig. 4D–F) in the enlarged perivascular and peribronchial areas (Fig. 4E) and in the alveolar spaces (Fig. 4F) in the lungs of mice already 6 days following exposure to sublethal dose of ricin in comparison to lungs of healthy mice (Fig. 4A–C). During the recovery phase, 30 days following exposure, the fibrotic process was more focal, but at the affected areas, formation of fibrous connective tissue was still noticeable (Fig. 4G), not only in the perivascular and peribronchial areas (Fig. 4H), but also in the alveolar interstitium (Fig. 4I).

**Figure 5:**
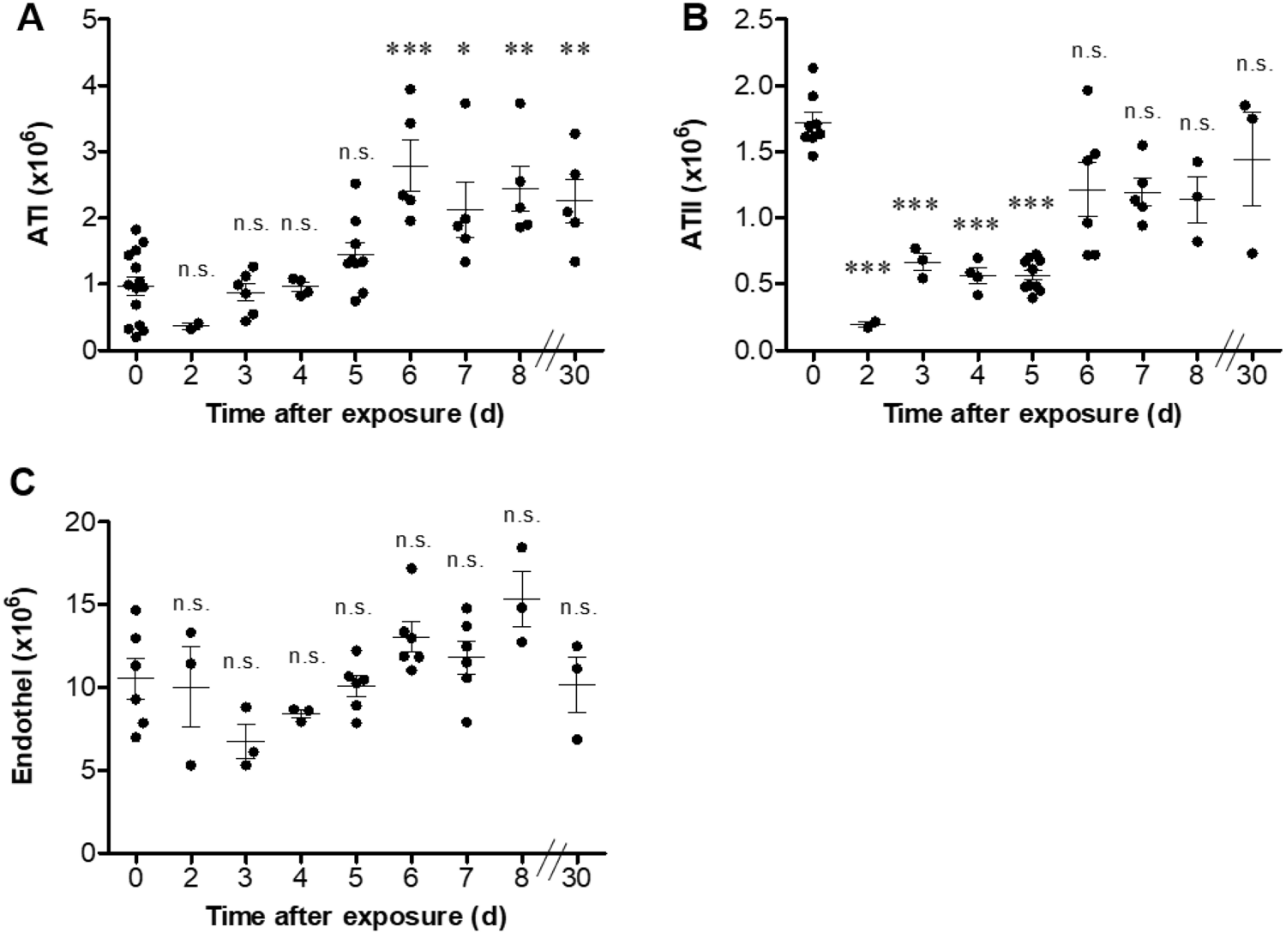
Parenchymal cell alterations following sublethal ricin intoxication. Lungs were isolated from intranasally ricin-intoxicated (1.7 µg/kg body weight) mice at indicated time-points. Lung cell suspensions were stained for (**A**) ATI, (**B**) ATII, (**C**) endothelial cells and analyzed by flow cytometry. Each dot represents single mouse. (*n* = *3–13*). Data are means ± SEM. *P < 0.05, **P < 0.01, ***P < 0.001, n.s., not significant in comparison to non-intoxicated mice.

Further analysis of the ricin-induced damage to the lung, following exposure to a sublethal dose, was performed by flow cytometry, which enabled the enumeration of lung parenchymal cells (Supplementary Fig. 3). Our analysis showed a significant increase in alveolar type I (ATI) epithelial cells from day 6 to 30 following exposure (Fig. 5A). In parallel, a transient reduction in alveolar type II (ATII) epithelial cells was observed during the first 5 days post-exposure. At later time-points, the number of ATII cells remained lower, yet not significantly different from their basal levels (Fig. 5B). We did not detect significant changes in the number of endothelial cells throughout the first 8 days following ricin intoxication, nor at day 30 post exposure (Fig. 5C).

### Inflammatory response and immune cell alterations in the lungs of mice following sublethal ricin intoxication

**Figure 6:**
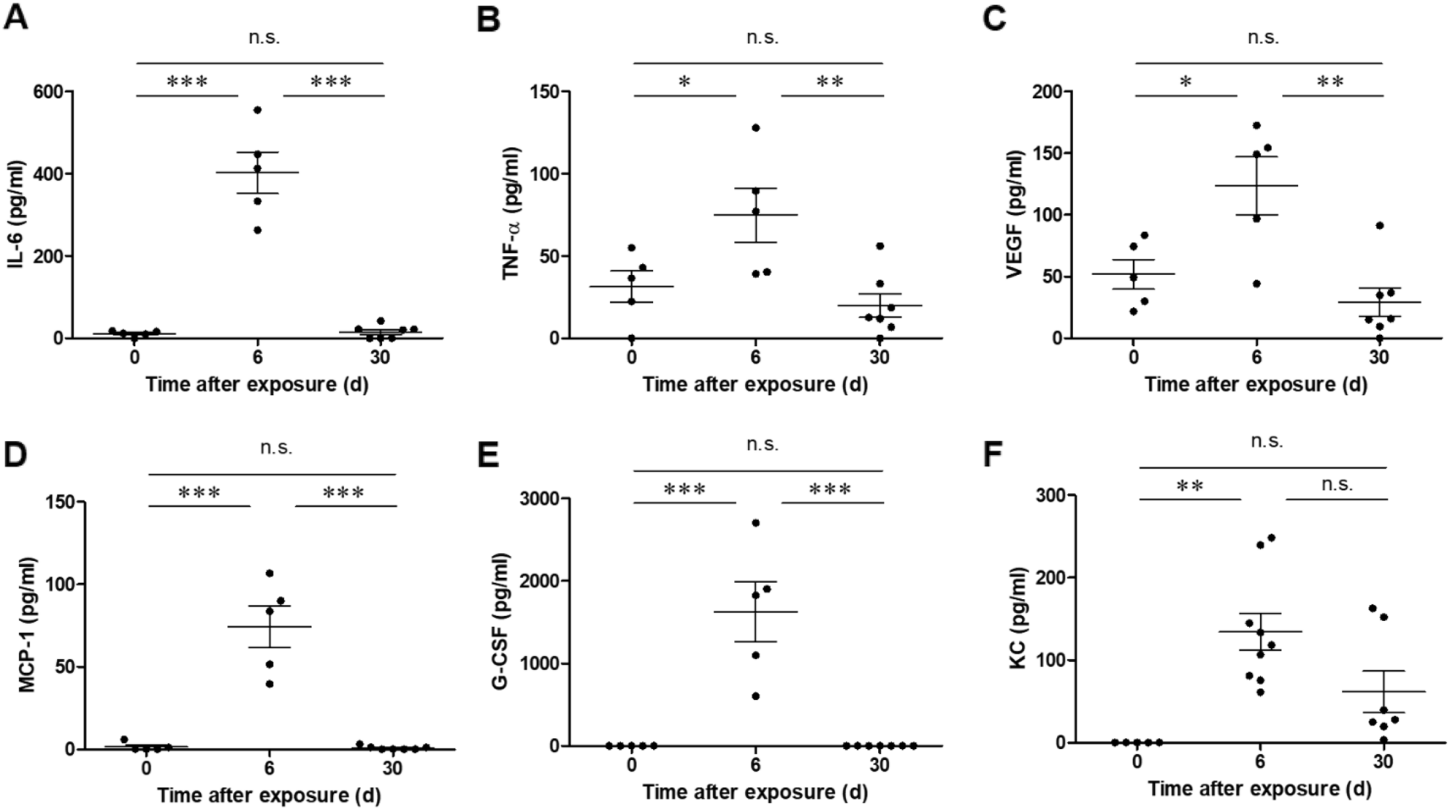
Pro-inflammatory cytokines in the BALF of mice following sublethal ricin intoxication. Mice were intranasally intoxicated with (1.7 µg/kg body weight) ricin and BALF samples collected at the indicated time-points were monitored for (**A**) IL-6, (**B**) TNF-α, (**C**) VEGF, (**D**) MCP-1, (**E**) G-CSF and (**F**) KC. Each dot represents single mouse; (*n* = *5–9*). Data are means ± SEM. *P < 0.05, **P < 0.01, ***P < 0.001, n.s., not significant.

One of the hallmarks of pulmonary exposure to ricin is the activation of a massive inflammatory response in the lungs^12,19,28,29^. We measured the levels of pro-inflammatory cytokines in bronchoalveolar lavage fluid (BALF) samples collected at the peak stage of the intoxication and at the recovery phase following sublethal intoxication. At day 6 after ricin exposure, we detected elevated levels of IL-6 (Fig. 6A), TNF-α (Fig. 6B) and VEGF (Fig. 6C). Increase in these proinflammatory cytokines levels correlated with acute phase response^30^, neutrophil-dependent vascular hyperpermeability^31^ and vascular permeability and interstitial edema^32^, respectively. At this time point, we also found that the levels of monocyte chemoattractant protein (MCP)-1 (Fig. 6D), granulocyte colony-stimulating factor (G-CSF) (Fig. 6E) and neutrophil chemoattractant KC (CXCL1) (Fig. 6F) were elevated. Importantly, at the recovery phase, 30 days post-exposure, all indicated cytokine levels were substantially reduced and their levels did not differ statistically from the levels in BALF of healthy mice (Fig. 6A–F). It should be mentioned that no change in the levels of MIP-2, IL-10 and IL-12 was detected (data not shown).

**Figure 7:**
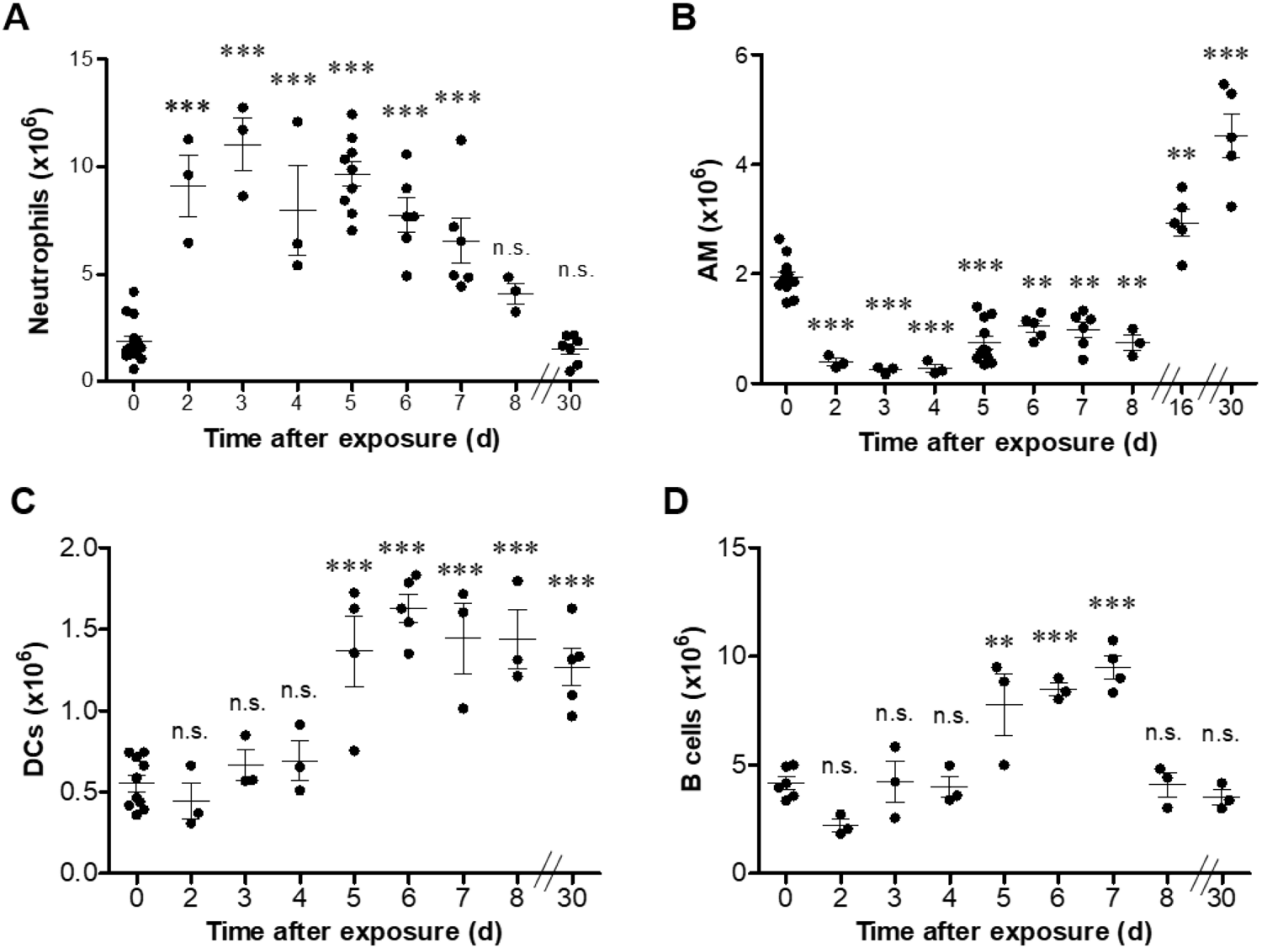
Characterization of immune cell populations in the lungs at different time-points following sublethal ricin intoxication. Lungs were isolated from intranasally ricin-intoxicated (1.7 µg/kg body weight) mice at indicated time-points. Cell suspensions were stained for (**A**) Neutrophils, (**B**) AM, (**C**) DCs, (**D**) B cells and analyzed by flow cytometry. Each dot represents single mouse. (*n* = *3–13*). Data are means ± SEM. *P < 0.05, **P < 0.01, ***P < 0.001, n.s., not significant in comparison to non-intoxicated mice.

Since the above analyzed chemoattractant cytokines affect the recruitment of cells to the lung and it has been previously published that pulmonary ricin intoxication induces death of dendritic cells, macrophages^8,33^ and in parallel, induces neutrophil influx to the lungs^5,8,13,15,23,28^, we performed a quantitative analysis of different cell populations in the lungs (Supplementary Fig. 3) during the first 8 days and at day 30 post exposure. Neutrophils were elevated till day 6 post-exposure, and then their numbers were gradually decreased. Already at day 8, the numbers did not differ significantly from the numbers acquired from healthy lungs (Fig. 7A). Alveolar macrophage (AM) number was significantly reduced already on day 1 post intoxication. This population quench remained during the following week. However, elevation above the normal numbers was observed at later time-points of 16- and 30-days post-exposure (Fig. 7B). A significant increase in dendritic cell (DCs) numbers (~ threefold) was detected only at day 5. This increment sustained during the following days until day 30 post-exposure (Fig. 7C). A transient elevation in B cell numbers was observed only on day 5 to day 7, before they returned to their basal levels on day 8 (Fig. 7D). It is worth mentioning that the significant activation of the immune response was also demonstrated by elevation in binding and neutralizing antibodies to ricin at day 30 post intoxication (Supplementary Fig. 4A,B).

## Discussion

In military or civilian scenarios involving ricin dissemination, the most likely outcome of an attack would be a large-scale poisoning at a low dose. The rationale behind this assumption is that the possibility of achieving a persistent lethal dose of ricin in unrestricted individuals in a relatively open environment would be less than achieving a sublethal dose^6^. The individuals exposed to a low dose will inevitably pose a substantial logistical burden on medical care systems. These individuals are also most likely to be aided by administration of medical countermeasure^21,34^. Several studies investigated the effects of inhalation exposure to a sublethal dose of ricin in rodents and non-human primates. The majority of these models examined mainly the acute phase following exposure to sublethal dose of ricin. They analyzed the lungs of the animals, mainly by histology and reported observations of alveolar edema, infiltration of inflammatory cells, hemorrhages, necrosis of pneumocytes and later, hyperplasia of epithelial cells, alveolar macrophage accumulation and moderate interstitial deposition of collagen, as part of fibrotic process^6,15,20–23^. To the best of our knowledge, only one study showed late pathological consequences of ricin intoxication in macaques. In this study, extensive fibrosis, infiltrates containing neutrophils and macrophages, including foamy macrophages within alveolar spaces were detected 20 days post exposure^10^. Recently, a new study evaluated the long-term pulmonary damage (30 days post-exposure) in surviving anti-ricin antibody-treated mice following a lethal ricin intoxication. This study showed evidence of lung fibrosis, hyperpermeability and decreased lung compliance^35^. In majority of these studies that aimed to understand the outcomes of sublethal exposure to ricin, the efforts were focused on the short-time period post exposure, the acute phase of the disease with merely evaluation of the long-term toxicity of ricin. Moreover, these studies did not perform correlation between lung pathology and evaluation of long-term physiological functionality of surviving animals.

In the current study we performed analysis of different physiological parameters in intoxicated mice. We monitored morbidity according to body weight loss, as well as by functional assessment of the animals as represented by the voluntary running activity and lung compliance. In addition to previous studies, we followed animal condition during both the acute phase and the recovery phase up to day 30 post exposure to sublethal ricin dose administered by the pulmonary route.

In agreement with the above-mentioned studies, our data represented similar lung pathology at the short-term period following sublethal pulmonary exposure of CD1 mice. In our study we used a sublethal dose of 0.35LD_50_ (1.7 µg/kg) by intranasal instillation. This sub-lethal intoxication dose is within the range that was used in other studies, using rodent models, where aerosolized inhalation or intratracheal instillation were used. We show here that the damage during the acute phase of our study recapitulates the damage caused by sublethal dose of ricin which was applied by aerosolized inhalation^15,20,21,23,34^. In the past, we determined ricin intranasal instillation LD_50_ of rodents in the range of 3.5–4.8 µg/kg^8,9,28,29,36^. Aerosolized inhalation LD_50_ in mice is somewhat higher and ranges from 10 to 20 µg/kg^15,20,21^. This may be explained by the anatomical structure of the mouse nasal turbinate, which filters a substantial amount of inhaled aerosolized particles^37^. LD_50_ in other animal models, such as Rhesus macaques require a relatively lower dose of ~ 6 µg/kg by aerosol inhalation^10,38–40^. Hence, we can assume that inhalation exposure LD_50_ per kg body in non-human primates corresponds with intranasal instillation LD_50_ per kg body in mice. There is a lack of documented studies that directly compare the differences in ricin deposition between inhalation and intranasal administration. However, research on lung deposition in other models has shown differences between aerosol inhalation exposure and intranasal instillation^41^. Despite the possible differences in toxin deposition, the overall damage in the lung following the two exposure routs is similar^11,34,42^. The damage to the lung that we observed during the early time points post-sublethal exposure, included marked interstitial pneumonia, neutrophil infiltration, pro-inflammatory cytokine response, alveolar macrophage and alveolar epithelia type II cell death and edema. These observations stand in line with previous studies, where a lethal dose was used by intranasal instillation^8,9,28,29,35,36^, which also corresponded with other studies in rodents following inhalation exposure^34,42,43^. Altogether, our findings further substantiate intranasal instillation as a solid alternative respiratory exposure route for ricin intoxication in mice.

In our current study, during the acute phase, mice lose approximately 20% of body weight, and lung hyperpermeability, neutrophil influx to the lungs, alveolar macrophage and epithelial type II cell depletion were detected. Histopathological analysis demonstrated widespread edema, cell infiltration, alveolar damage, bronchiolitis and inflammation. Moreover, we observed during the acute phase, increased pro-inflammatory response, as indicated by elevation of IL-6, TNF-α, MCP-1, G-CSF and KC. Our previous findings have shown an increased inflammatory response in the lungs at earlier time-points following exposure, as evidenced by elevated cytokine protein levels in the BALF 48 h after sublethal ricin intoxication^24^ and in the cytokine mRNA levels of mice 96 h post sublethal ricin exposure^44^. Some of these cytokines are responsible for the observed influx of neutrophils into to the lungs, which in turn with their toxic mediators cause tissue injury, including an increase in lung epithelial and endothelial permeability^45^. Indeed, significant elevation in the levels of CXCL1 (KC), CXCL2 (MIP-2)^46^ and CCL2 (MCP-1), a classic chemoattractants for monocytes, contribute to neutrophil recruitment in acute lung injury^47^. G-CSF secreted during acute inflammation provides survival signals to neutrophils and enhances their half-live^48^. VEGF and TNF-α secreted in the lungs, lead to increased endothelial permeability^49,50^ and impaired alveolar fluid clearance^51^, which would explain the lung hyperpermeability and edema during the acute phase of the disease. In agreement with our data on secreted cytokines, studies on sublethal ricin exposed lung tissue in mice identified increased expression of genes, coding for proteins responsible for pro-inflammatory response, formation of endothelial gaps, maintenance of membrane integrity and cellular adhesion, apoptosis and early healing response^15,21,22^. The extent of lung injury following sublethal exposure to ricin was also evaluated by examination of lung morphology and cellular changes. All of these parameters pointed to severe short-term damage to cellular composition lung architecture. The damaged lung tissue caused impairment in lung compliance, which led to physical morbidities, such as weight loss and substantial decrease in voluntary activity. Pulmonary compliance, a measure of the expansion of the lung, is critical to the proper function of the respiratory system. Factors affecting pulmonary compliance include elasticity of the tissue and surface tension, which is decreased by surfactant production. Alveolar epithelial type II cells secrete pulmonary surfactant, which ultimately decreases alveolar surface tension to prevent alveolar collapse, as well as decreasing elastance and increasing compliance^52^. Thus, the decreased pulmonary compliance of intoxicated mice may be attributed to diminished number of alveolar epithelial type II cells and consequently, reduced surfactant levels. Interestingly, certain parameters were found to be maximally affected as early as 48 h post-exposure, preceding the time-point of maximal morbidity on day 6. For instance, the elevation in neutrophils and reduction in alveolar epithelial type II cells were prominent at 48 h, while the reduction in alveolar macrophages and decreased nocturnal activity were similarly affected at both 48 h and day 6 post-exposure.

Gal et al*.* demonstrated that mice surviving lethal ricin intoxication following anti-ricin antibody treatment exhibited an impaired pulmonary compliance. However, when ciprofloxacin was co-administered with the anti-ricin antibody treatment, lung function in these mice was restored^35^. Based on these findings, we suggest that ciprofloxacin or doxycycline treatment of mice exposed to sublethal doses of ricin may reduce pro-inflammatory cytokine response, lower markers of oxidative stress, mitigate hyperpermeability of the lungs^28^ and improve pulmonary compliance^35^.

The aberrant function of the respiratory system had a direct effect on the voluntary activity of the mice. Immediately following ricin intoxication, running activity decreased rapidly for few days. These data indicated that during the acute phase of the disease, mice were heavily affected by the toxin, experiencing substantial morbidity.

Analysis of the outcomes in the long-term, during the recovery phase of the disease, demonstrated considerable improvement of the lung injury. This alleviation was evidenced by ceased pro-inflammatory response, repair of alveolar epithelial type II cells, recovery of lung compliance and normal voluntary activity. Notably, exposure even to a higher sublethal dose of ricin did not result in aberrant lung compliance through the recovery phase. Nevertheless, there was still some evidence of edema and hyperpermeability of the lung, indicated by histology and lung/body weight (%) ratio. Despite the adequate function of the respiratory system and mouse voluntary running activity, lung histopathological abnormalities were still present at 30 days post-ricin exposure. Confined pulmonary lesions included edematous and hemorrhagic areas, and peribronchovascular and alveolar fibrosis. However, we did not observe ATII hyperplasia, an indicative of alveolar epithelial repair. The lack of hyperplasia in the alveoli could be a consequence of the low challenge dose, as was reported previously^21^. Following ricin intoxication, fibrosis was prominent around terminal respiratory bronchioles, in the interstitium surrounding large blood vessels and extending into alveolar septa, similar to other reports^6,10,20^. In addition, we report here that surrounding the areas of fibrosis and within the alveolar spaces, there is accumulation of large numbers of foamy macrophages (alveolar histiocytosis). Generally, the fibrosing interstitial pneumonia and type II pneumocyte hyperplasia with minimal edema and alveolar histiocytosis are indicative of an ongoing reparative process. The initial injury during the acute phase transforms into a recovery phase involving an influx of macrophages, clearing the alveolar spaces and repairing the pulmonary architecture by robust fibrotic response^6^. These repair mechanisms lead to restoration of the lung and relief of the clinical signs, although pathologically it seems that the lungs did not return to a completely normal state, due to fibrosis. Accordingly, we predict that sublethal doses of aerosolized ricin will result in recovery from acute lung disease, but surviving individuals might develop signs of long-term fibrosis at varying severities. Therefore, we suggest that the use of ciprofloxacin or doxycycline alone, could be effective in mitigating the respiratory complications associated with sublethal ricin exposure in the short-term. These treatments may help restore normal lung function and minimize the subsequent fibrosis. In addition, targeted medicament for prevention of lung fibrosis, such as pirfenidone and nintedanib, which were proposed for treatment of pulmonary fibrosis developed upon SARS ARDS^53^, should be considered.

## Materials and methods

### Animal ethics

All animal experiments were performed in accordance with relevant guidelines and regulations by the Israeli law and approved by the Institutional Animal Care and Use Committee (IACUCs) at the Israel Institute for Biological Research (protocol number M-42-2021 (body weight changes, wet/dry lung weight and lung /body weight ratios, Evans Blue extravasation assay, flow cytometry of lung cells, lung compliance); M-49-2021 (nocturnal running on the wheel, lung histology); M-54-2021 (lung compliance); M-06-2021 (flow cytometry of lung cells), M-36-2022 (partial lethal dose, survival and body weight changes, lung compliance) and followed by the ARRIVE guidelines (https://arriveguidelines.org). Treatment of animals was in accordance with regulations outlined by the USDA Animal Welfare Act and the conditions specified by the National Institute of Health Guide for Care and Use of Laboratory Animals. During intoxication experiments animals were monitored daily. Mice that exhibited two of the following signs: matted hair, a hunched posture, breathing difficulty, and tremors were considered for euthanasia. Notably, body weight loss was not used as an end-point criterion, as mice can fully recover and regain their pre-poisoning weight even after losing up to 30% of their initial body weight.

### Animals

Female CD-1 mice (27–32 g) were purchased from Charles River Laboratories Ltd. (Margate, UK). Mice were housed in filter-top cages in an environmentally controlled room and maintained at 21 ± 2 °C and 55 ± 10% humidity. Lighting was set to mimic a 12/12 h dawn to dusk cycle. Mice were housed in a purpose-built animal holding facility for 4–8 days prior to the beginning of the experiment. Animals were allowed access to water and food ad libitum.

### Ricin preparation and intoxication

The use of ricin in the current study and purification of ricin from castor beans were conducted under the safety and environmental regulations of the Israel Institute for Biological Research, in compliance with the Israeli law.

Crude ricin was prepared from seeds of endemic *Ricinus communis* as previously described^28^. Shortly, seeds were homogenized in a blender (Waring, Torrington, CT) in 5% acetic acid (Merck, Darmastadt, Germany)/PBS (Biological Industries, Beth-Haemek, Israel). The homogenate was centrifuged, and the clarified supernatant containing the toxin was subjected to ammonium sulfate (Merck, Darmastadt, Germany) precipitation (60% saturation). The precipitate was dissolved in PBS and dialyzed extensively against the same buffer. The toxin preparation appeared on a Coomassie blue (Bio-Rad, Rishon Le Zion, Israel)-stained nonreducing 10% polyacrylamide gel (ThermoFisher Scientific, Carlsbad, CA) as two major bands of molecular mass of ~ 65 kDa (ricin toxin, ~ 80%) and 120 kDa (*Ricinus communis* agglutinin, ~ 20%). Protein concentration was determined as 2.86 mg/ml by 280-nm absorption (Nanodrop 2000; ThermoFisher Scientific).

Prior to intoxication, mice were anesthetized by an intraperitoneal injection of ketamine (1.9 mg/mouse, Vetoquinol, Lure, France) and xylazine (0.19 mg/mouse, Eurovet Animal Health, AD Bladel, The Netherlands). Crude ricin at a dose of 0.35 or 0.5 × median lethal dose (0.35LD50, 1.7 µg/kg or 0.5LD50, 2.4 µg/kg body weight, 50 µl) was applied intranasally.

### ELISA for ricin binding antibodies

Microtiter plates coated with pure ricin^28^ at concentration 2.5 µg/ml diluted in Carbonate-bi-carbonate buffer 50 mM, pH 9.6 (Sigma, C30450, Israel) were incubated over night at 4 °C. Then, the plates were blocked using PBT buffer (2% BSA, 0.05% Tween 20, 0.05% Na-Azide in PBS) for 1 h at 37 °C. Next, serum samples at the initial dilution of 1:10 in PBT buffer were added to the plate and further twofold dilutions were performed. Plates were incubated for 1 h at 37 °C, washed and reacted with anti-mouse whole IgG-AP (Sigma, A1902, Israel) and incubated for 20 min at 37 °C. Detection was performed using PNPP substrate (Sigma, N1891, Israel). The reaction was measured at 405 and 620 nm in a Spectramax ABS (Molecular Devices, Sunnyvale, CA, USA).

### In vitro ricin neutralization assay

The level of ricin neutralizing antibodies in mice sera was measured in cell culture by assessment of inhibited expression of ubiquitin luciferase enzyme, as previously described^54^. HeLa Ub-FL cells (stably expressing ubiquitin-luciferase^55^) were a kind gift from Professor Piwnica-Worms (University of Texas, MD Anderson Canser Center, USA). Cells cultured in DMEM (Biological Industries, Beit Haemek, Israel) supplemented with 10% FCS were seeded in 96-well plates (3 × 10^4^ cells/well), and 24 h later the medium was replaced by culture medium containing 10 ng/ml ricin in presence of increasing dilutions of mouse serum. One day later, the medium was replaced for fresh medium supplemented with 10 µM of MG132 proteasome inhibitor (Sigma, C2211, Israel) for 1 h. Next, the medium was replaced by 50 µl of lysis buffer (Promega, E1941, Israel) for 10 min at RT. The residual luciferase activity in each well was determined by mixing equal volumes of the cell lysate and Luciferase assay reagent (Promega, E1483, Israel) followed by immediate measurement of luminescence levels using Victor^3^ plate reader (Perkin-Elmer, Shelton, CT, USA). Neutralization antibody titer was determined using GraphPad Prism software (version 5.01, GraphPad Software Inc., La Jolla, CA, USA, 2007).

### Lung permeability analysis

Lung permeability was determined by the Evans Blue dye (EBD) extravasation method as follows: EBD (7.5 mg/ml, Sigma-Aldrich, Rehovot, Israel) was injected intravenously into mice at a dose of 50 mg/kg, and allowed to circulate for 1 h. Mice were then anesthetized, and the lungs were perfused by cutting the left atrium and flushing with 5 ml PBS through the right ventricle. The lungs were removed and EBD was extracted by incubation of the tissues in 0.5 ml of formamide (Sigma-Aldrich, Israel) at 60 °C for 24 h. EBD optical density in the supernatant was measured at 620 nm by spectrophotometer (Molecular Devices, Sunnyvale, CA, USA) and the total amount of dye was calculated by means of a standard calibration curve. For wet/dry lung weight analysis, the lungs were removed, weighed, dried at 60 °C for 5 days and reweighed.

### Histology

Lungs were collected and fixed in 4% buffered formaldehyde in PBS pH 7.2–7.4 (Bio Lab, Israel) for 2 weeks. Sections of 5 µm were prepared after paraffin embedding using a microtome (RM 2255, Leica, Germany) and stained with Hematoxylin and Eosin (H&E). For fibrosis visualization, Masson’s Trichrome (Sigma, Israel) staining was performed according to the manufacturer’s instructions. Blue-stained areas (aside of the blue staining of vascular structures) corresponded to fibrotic areas. Images were captured with a slide scanner 3D HISTECH Panoramic Midi II slide viewer (Budapest, Hungary) and analyzed with Case Viewer (version 2.4.0, Budapest, Hungary).

### Inflammatory mediators

Bronchoalveolar Lavage Fluids (BALF) were collected by instillation of 1 ml PBS at room temperature via a tracheal cannula, and centrifuged at 240 g at 4 °C for 5 min. Supernatants were collected and stored at −20 °C until further use. Interleukin-6 (IL-6), tumor necrosis factor-α (TNF-α), vascular endothelial growth factor (VEGF), monocyte chemoattractant protein (MCP-1), granulocyte colony-stimulating factor (G-CSF) and keratinocyte chemoattractant (KC) were quantified using ELISA kits (R&D Systems, USA), following the manufacturer’s instructions.

### Flow cytometry

Lungs were harvested, cut into small pieces and digested as follows: for analysis of hematopoietic cells tissue was treated 2 h at 37 °C with 4 mg/ml collagenase D (Roche, Mannheim, Germany) in PBS Ca^+2^ Mg^+2^ (Biological Industries, Beit Haemek, Israel); for analysis of parenchymal cells tissue was treated 1 h at 37 °C with 1.7 mg/ml collagenase A, 5 U/ml Dispase II, 0.3 mg/ml DNase I (all Roche, Mannheim, Germany) in RPMI medium (Biological Industries, Beit Haemek, Israel). The tissue was then meshed through a 40 μm cell strainer and red blood cells were lysed with red blood cell lysis buffer (Sigma-Aldrich, Rehovot, Israel). Cell suspensions were stained with the following fluorophore conjugated antibodies: CD45 (clone 30-F11), CD11c (N418), MHC class II (M5/114.15.2), Ly6G (1A8), CD170 (S17007L), CD11b (M1/70), CD19 (6D5), CD31 (390), CD326 (G8.8), Podoplanin (8.1.1). Antibodies were purchased from BioLegend, BD Biosciences or eBioscience. For dead cell exclusion, Aqua Live/Dead cell stain (ThermoFisher) was used. Cells were collected by flow cytometry using LSR-Fortessa (BD Biosciences, San Jose, CA, USA) and analyzed by FlowJo software (version 10, Tree Star, Ashland, OR, USA).

### Lung compliance

Lung compliance was measured using an inhouse-made device. Two plastic tubes were inserted to a T-splitter connector, one of which was connected to a cannula, and the other one to a monometer (a U-shape tube filled with colored liquid). Mice were euthanized, tracheotomized and the cannula (26GA, 0.6 × 19 mm, BD Neoflon, Singapore) was inserted to the trachea, above the bifurcation. A plastic syringe was connected to the T-splitter and 1 ml air was ventilated into the apparatus and calculation of compliance was calculated as follows: compliance = ΔV/ΔP where ΔV is the volume of air ventilated (1 ml) and ΔP is the change in pressure as reflected by the increase in water meniscus of the monometer. The shift (rise) in liquid meniscus was inversely correlated to lung compliance.

### Mouse activity measurement

Mouse activity was measured using a running-wheel monitoring system as previously described^25^. Briefly, prior to ricin intoxication, the animals were gradually acclimated to voluntary running. Initially, 4 mice were housed per cage, with access to 2 running wheels for about 7 days. Afterwards, mice were split into running-monitored cages, with one mouse per cage, for at least 5 days. During these days, individual voluntary running distance was monitored for consistency (relative standard deviation (RSD) < 30%). Mice showing consistent and robust running (at least 6 km/night) were ricin intoxicated and monitored for 30 days.

### Statistical analysis

All statistical analyses were conducted with GraphPad Prism software (version 5.01, GraphPad Software Inc., La Jolla, CA, USA, 2007). Data is presented as means ± SEM. For multiple comparisons, one-way analysis of variance (ANOVA) tests followed by Tukey’s multiple comparisons test were applied. Differences were considered significant at *p* < 0.05.

### Supplementary Information

Supplementary Figures.

## Data Availability

The data that support the findings of this study are available on reasonable request from the corresponding authors.
