# Supplementary material for: Short- and long-term outcomes of pulmonary exposure to a sublethal dose of ricin in mice
Source: Sci Rep. 2024 May 21;14:11637. doi: 10.1038/s41598-024-62222-9 (PMC11109263; doi:10.1038/s41598-024-62222-9)
Supplement: Supplementary file 1 — Supplementary Figures. [file 41598_2024_62222_MOESM1_ESM.pdf]

Supplementary Figures

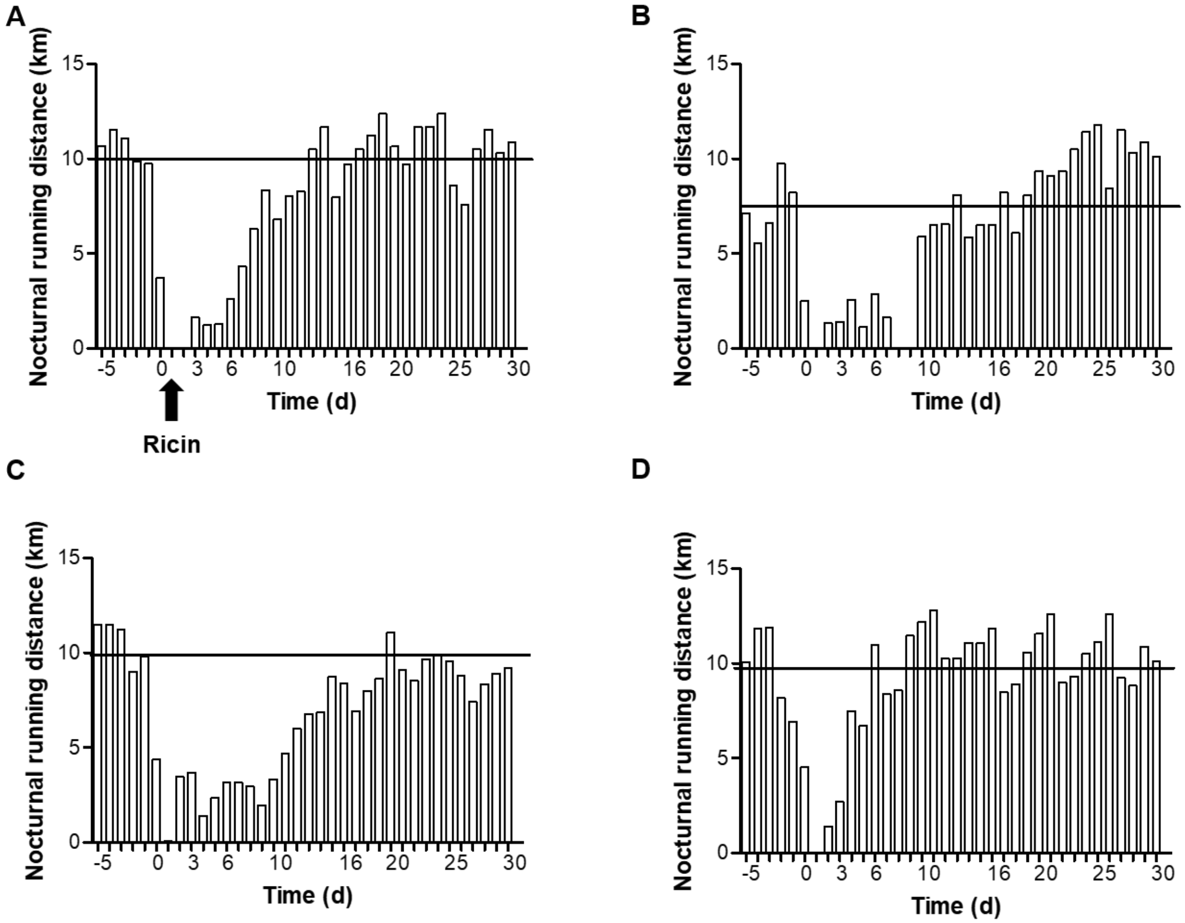

Supplementary Figure 1. Activity profile of individual mice according to voluntary wheel running following sublethal exposure to ricin toxin. The activity was recorded 5 days before intoxication for receiving consistent activity of each healthy mouse. Then, the mice were exposed to ricin and their running was further recorded for 30 days following intoxication. (**A,B,C,D**) Representative activity profile of four different mice. Horizontal line indicated the average running distance of the mouse prior to intoxication. The time-point 0 represented ricin intoxication.

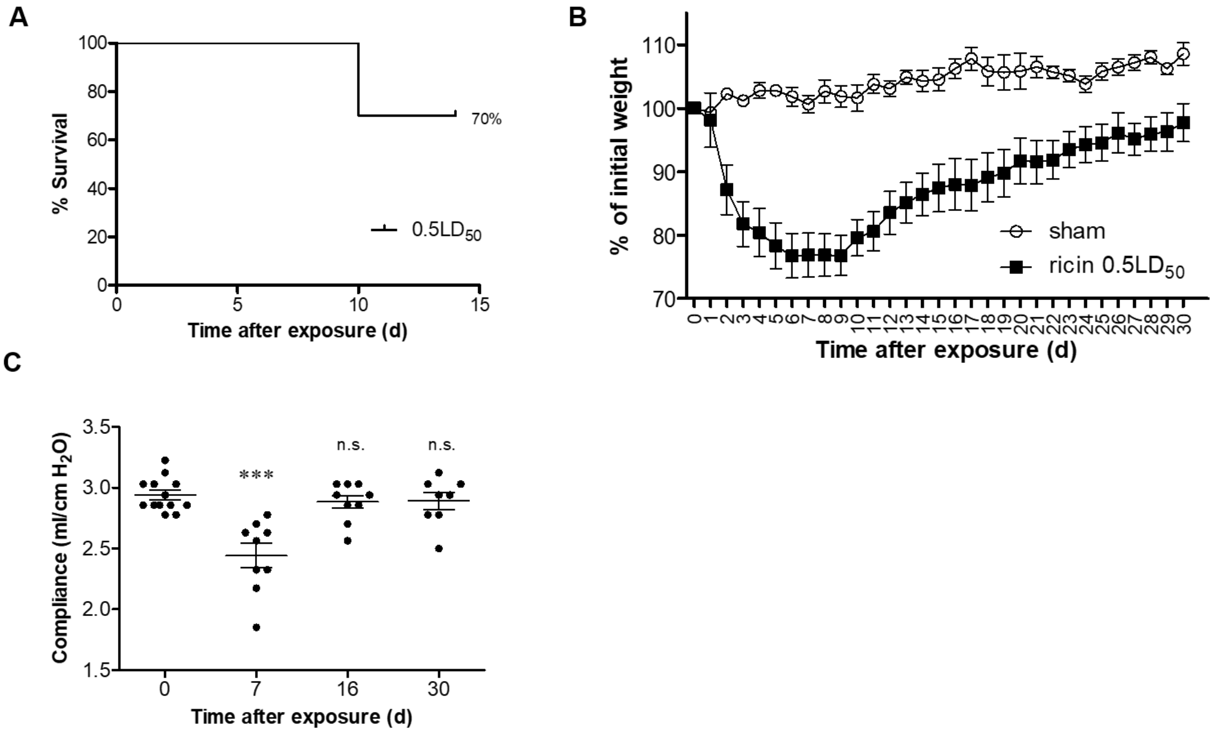

Supplementary Figure 2. Physiological and functional changes in mice following exposure to partially lethal dose of ricin toxin. Mice were intranasally intoxicated with $0.5LD_{50}$ (2.4 µg/kg body weight) ricin and monitored for morbidity. (**A**) Survival (*n=10*), (**B**) body weight loss (*n=5-7*) and (**C**) lung compliance (*n=8-13*). Each dot represents one animal. Data are represented as means ± SEM. $^{***}P < 0.0001$, n.s., not significant, in comparison to non-intoxicated mice.

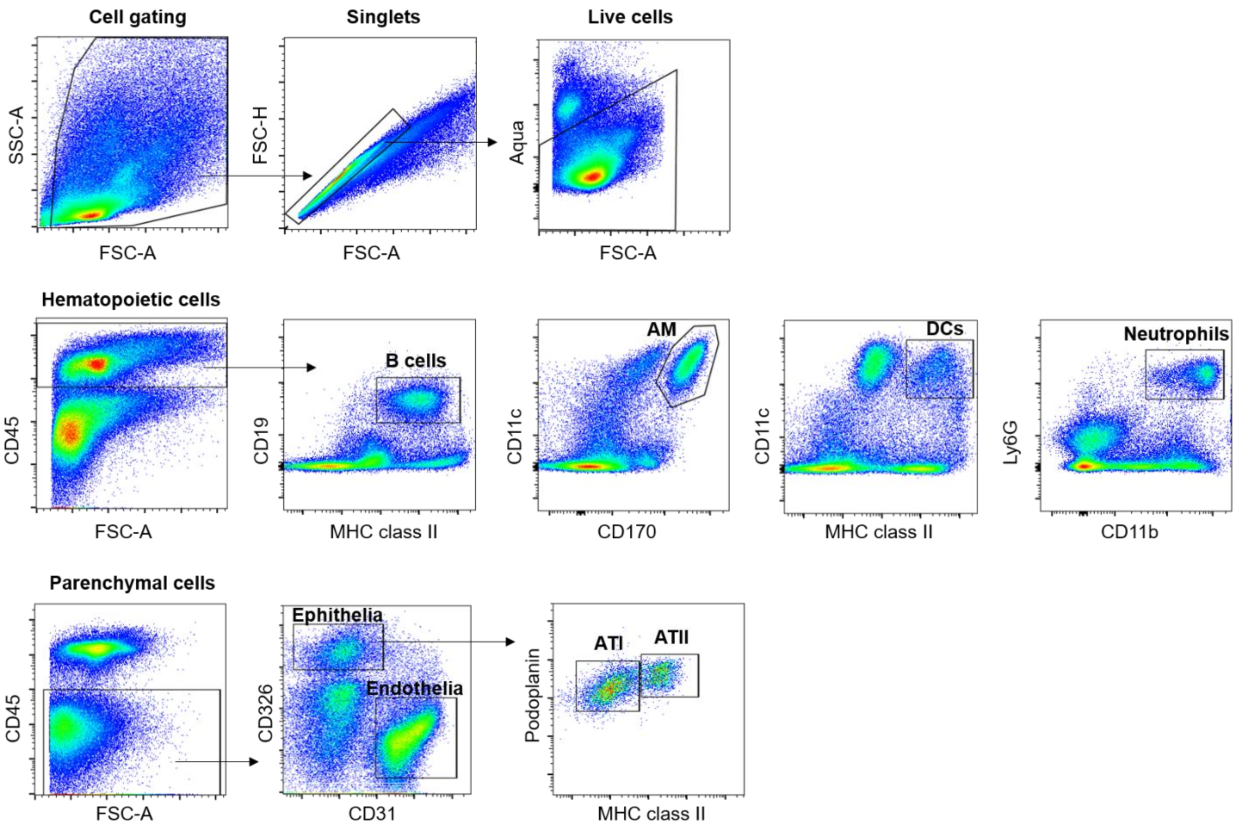

Supplementary Figure 3. Gating strategy to identify differential cell populations in the lungs. Cells were gated for intact singlets and live (Aqua^{neg} cells) cells. For hematopoietic cell identification, cells were gated for CD45^{high} cells. B cells were identified as CD19^{high} and MHC class II^{high} cells, alveolar macrophages (AM) as CD11c^{high} and CD170^{high}, dendritic cells (DCs) as CD11c^{high}, CD170^{low} and MHC class II^{high} and neutrophils as Ly6G^{high} and CD11b^{high}. Lung parenchymal cells were first defined as CD45^{neg} cells. Then, endothelial and epithelial cells were gated as CD326^{neg}, CD31^{high} and CD326^{high}, CD31^{neg}, respectively. Epithelial cells were segregated into alveolar epithelial type I (ATI) and alveolar epithelial type II (ATII) cells by gating Podoplanin^{high}, MHC class II^{low} and Podoplanin^{high}, MHC class II^{high} cells[43], respectively.

A

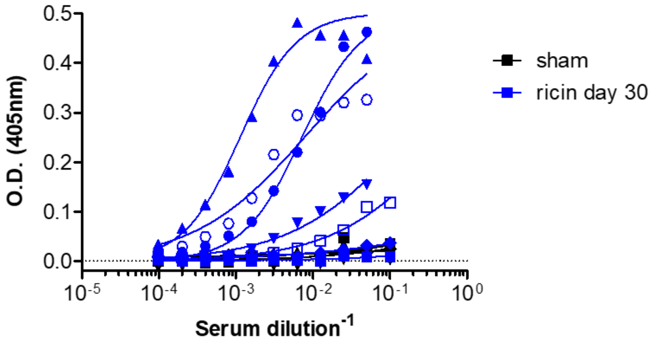

B

Serum	Binding (DIL_{50})	Neutralization (NT_{50})
1 – day 30	139	9
2 – day 30	881	9
3 – day 30	128	4
4 – day 30	6	0
5 – day 30	2	0
6 – day 30	0	0
7 – day 30	0	0

Supplementary Figure 4. Development of intrinsic antibody response in mice following sublethal exposure to ricin toxin. Mice were intranasally intoxicated with (1.7 μg/kg body weight) ricin and their sera were characterized. (**A**) The sera at various dilutions were tested for presence of ricin-binding antibodies by ELISA. Each curve represents single mouse. Black line, sham; blue line, serum of mice at day 30 after ricin intoxication; (*n*=*5*-*7*). (**B**) Summary of antibody titer (DIL_{50}) and neutralization potency (NT_{50}) values from serum of mice 30 days after ricin exposure, (*n*=*7*).
